# Engineering transcription factors to improve tolerance against alkane biofuels in *Saccharomyces cerevisiae*

**DOI:** 10.1186/s13068-015-0411-z

**Published:** 2015-12-30

**Authors:** Hua Ling, Nina Kurniasih Pratomo Juwono, Wei Suong Teo, Ruirui Liu, Susanna Su Jan Leong, Matthew Wook Chang

**Affiliations:** Department of Biochemistry, Yong Loo Lin School of Medicine, National University of Singapore, 8 Medical Drive, 117597 Singapore, Singapore; NUS Synthetic Biology for Clinical and Technological Innovation (SynCTI), Life Sciences Institute, National University of Singapore, 28 Medical Drive, 117456 Singapore, Singapore; Singapore Institute of Technology, 10 Dover Drive, 138683 Singapore, Singapore

**Keywords:** Pleiotropic drug resistance, Transcription factors, Site mutagenesis, Alkanes, Biofuels, Tolerance, *Saccharomyces cerevisiae*

## Abstract

**Background:**

Biologically produced alkanes can be used as ‘drop in’ to existing transportation infrastructure as alkanes are important components of gasoline and jet fuels. Despite the reported microbial production of alkanes, the toxicity of alkanes to microbial hosts could pose a bottleneck for high productivity. In this study, we aimed to improve the tolerance of *Saccharomyces cerevisiae*, a model eukaryotic host of industrial significance, to alkane biofuels.

**Results:**

To increase alkane tolerance in *S. cerevisiae*, we sought to exploit the pleiotropic drug resistance (Pdr) transcription factors Pdr1p and Pdr3p, which are master regulators of genes with pleiotropic drug resistance elements (PDREs)-containing upstream sequences. Wild-type and site-mutated Pdr1p and Pdr3p were expressed in *S. cerevisiae* BY4741 *pdr1Δ pdr3Δ* (BYL13). The point mutations of *PDR1* (F815S) and *PDR3* (Y276H) in BYL13 resulted in the highest tolerance to C10 alkane, and the expression of wild-type *PDR3* in BYL13 led to the highest tolerance to C11 alkane. To identify and verify the correlation between the Pdr transcription factors and tolerance improvement, we analyzed the expression patterns of genes regulated by the Pdr transcription factors in the most tolerant strains against C10 and C11 alkanes. Quantitative PCR results showed that the Pdr transcription factors differentially regulated genes associated with multi-drug resistance, stress responses, and membrane modifications, suggesting different extents of intracellular alkane levels, reactive oxygen species (ROS) production and membrane integrity. We further showed that (i) the expression of Pdr1_mt1_ + Pdr3_mt_ reduced intracellular C10 alkane by 67 % and ROS by 53 %, and significantly alleviated membrane damage; and (ii) the expression of the Pdr3_wt_ reduced intracellular C11 alkane by 72 % and ROS by 21 %. Alkane transport assays also revealed that the reduction of alkane accumulation was due to higher export (C10 and C11 alkanes) and lower import (C11 alkane).

**Conclusions:**

We improved yeast’s tolerance to alkane biofuels by modulating the expression of the wild-type and site-mutated Pdr1p and Pdr3p, and extensively identified the correlation between Pdr transcription factors and tolerance improvement by analyzing gene patterns, alkane transport, ROS, and membrane integrity. These findings provide valuable insights into manipulating transcription factors in yeast for improved alkane tolerance and productivity.

**Electronic supplementary material:**

The online version of this article (doi:10.1186/s13068-015-0411-z) contains supplementary material, which is available to authorized users.

## Background

Biologically synthesized alkanes can be used as ‘drop in’ to existing transportation infrastructure as alkanes are important components of gasoline and jet fuels [1]. Even though alkanes have been successfully produced in microbes [2–8], the yields and titers should be a key consideration for industrial-scale production, and the toxicity of alkanes to microbial hosts could eventually be a bottleneck for high productivity [9, 10].

Our previous transcriptome analyses suggested that alkanes induce a range of cellular mechanisms such as efflux pumps, membrane modification, radical detoxification, and energy supply in yeast [9]. Indeed, the mechanisms underlying cell responses to toxic molecules can provide useful strategies to improve cell tolerance and viability. Such strategies include engineering efflux pumps [9–12] and transcription factors [13–17], and modifying cellular membrane [18]. Transcription factors regulate multiple and simultaneous perturbations of the transcriptome towards a global phenotype of tolerance [19]. By knockout or overexpression of transcription factors involved in genetic regulatory networks of isooctane response in *Escherichia coli*, Kang et al. [20] improved *E. coli’s* tolerance to isooctane. In addition, Matsui et al. discovered a modified transcription factor endowing *Saccharomyces cerevisiae* with organic-solvent tolerance [21].

Towards the development of alkane-tolerant *S. cerevisiae*, a well-studied model eukaryote with wide industrial applications, we sought to exploit its transcription factors Pdr1p and Pdr3p, which are master regulators of genes with pleiotropic drug resistance elements (PDREs)-containing upstream sequences [22]. Currently, a thorough investigation of the roles of Pdr1p and Pdr3p in cellular tolerance to alkanes is lacking. In this study, we demonstrated a significant improvement in yeast’s tolerance to n-decane (C10) and n-undecane (C11) by modulating the expression of wild-type and site-mutated Pdr1p and Pdr3p. The correlation between Pdr transcription factors and tolerance improvement was confirmed by analyzing gene patterns, alkane transport, reactive oxygen species (ROS) levels, and membrane integrity.

## Results and discussion

### Site-mutation of *PDR1* and *PDR3*

**Fig. 1:**
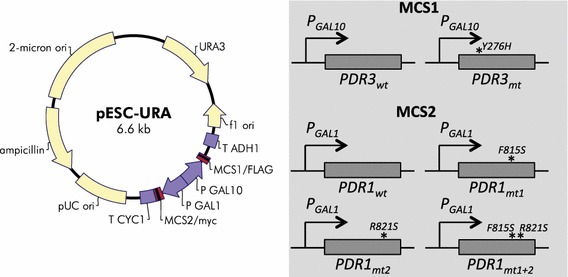
Site mutagenesis of *PDR1* and *PDR3*, and plasmid construction. Plasmid pESC-Ura (http://www.chem-agilent.com) was used as a vector to express the transcription factors. Wild-type and mutant alleles of *PDR1* and *PDR3* were cloned into MCS2 and MCS1, respectively. *Asterisks* mutation sites

Transcription factor engineering is widely used to improve microbial strain tolerance against toxic molecules [12, 14]. In *S. cerevisiae*, transcription factors Pdr1p and Pdr3p have a DNA-binding domain, an inhibitory domain, and a transcription activation domain. The inhibitory domain in a locked conformation interacts with the transcription activation domain [23, 24], which is associated with Pdr-DNA or Pdr–Pdr interactions and pleiotropic drug resistance. Amino acid substitutions in the inhibitory domains could alter the actions of the transcription activation domain, leading to changes in Pdr1 and Pdr3 activity and the pleiotropic drug resistance. Recently, a series of site mutations in the inhibitory domains have been shown to improve pleiotropic drug resistance, and three site mutations (F815S and R821S in Pdr1p, and Y276H in Pdr3p) are most effective to improve the tolerance against various toxic molecules [21, 23–25]. Currently, a thorough investigation of the roles of these mutations in cellular tolerance to alkanes is lacking. In this study, we chose F815S and R821S in Pdr1p and Y276H in Pdr3p for improving the tolerance to alkane biofuels in *S. cerevisiae*. Figure 1 shows the chosen mutation sites and cloning of the wild-type and site-mutated *PDR1* and *PDR3* into pESC-Ura. We induced the expression of the wild-type and site-mutated *PDR1* and *PDR3* in a double gene-deletion mutant *S. cerevisiae* BYL13 (*pdr1Δ pdr3Δ*).

### Conditions for protein induction and alkane exposure

To test yeast cell tolerance towards alkanes, we determined suitable conditions for protein induction and alkane exposure. Additional file 1: Figure S1A shows that the growth of BYL13 expressing the site-mutated Pdr transcription factor genes was inhibited, suggesting that lower induction of the Pdr transcription factors might lead to lower growth inhibition. To determine suitable induction conditions, we added various amounts of galactose (0.5 g/l, 5 g/l, and 20 g/l) and compared the resulting cell densities. Additional file 1: Figure S1B shows that the growth inhibition was lower (one-tailed Student *t* test, p < 0.05) at 24 h when the expression was induced by a lower concentration of galactose (0.5 g/l). Hence, we chose to use 0.5 g/l of galactose for the induction of transcription factor expression in further experiments.

Medium chain alkanes (C8–C12) are important components of transportation fuels such as gasoline and jet fuels. As C12 alkane exhibits insignificant toxicity to *S. cerevisiae* [10], we aimed to improve yeast tolerance towards C8, C9, C10, and C11 alkanes. Additional file 1: Figure S2 shows that 5 % of C8, C9, or C11 alkanes, and 1 % of C10 alkane inhibited the growth of BYL13 carrying empty pESC-Ura plasmid. These alkane concentrations, together with 0.5 g/l of galactose, were used for determining engineered yeast tolerance towards alkanes.

### Tolerance of BYL13 expressing Pdr transcription factors towards alkanes

**Fig. 2:**
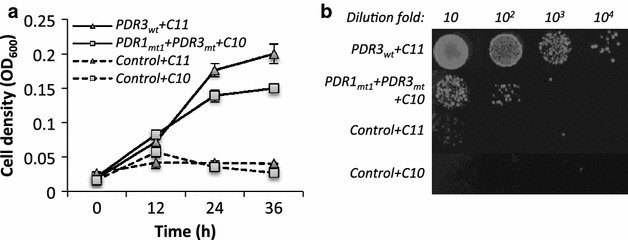
Tolerance of BYL13 + Pdr (Pdr1_mt1_ + Pdr3_mt_ and Pdr3_wt_) against C10 and C11 alkanes. **a** Cell density (OD_600_) of BYL13 expressing Pdr1_mt1_ + Pdr3_mt_ against 1 % C10 (PDR1_mt1_ + PDR3_mt_ + C10), and BYL13 expressing Pdr3_wt_ against 5 % C11 (PDR3_wt_ + C11) was measured every 12 h. *Error bars* SD from three biological replicates. **b** Ten microliters of serially diluted cells (24 h) were spotted onto YPD agar plates for cell viability assays. Control, BYL13 with pESC-Ura

We then investigated the tolerance of BYL13 expressing Pdr transcription factors against C8, C9, C10, and C11 alkanes. Figure 2a and Additional file 1: Figure S3 show that in the presence of (i) 1 % C10 alkane, BYL13 expressing the site-mutated Pdr transcription factors (particularly Pdr1_mt1_ + Pdr3_mt_) had significantly higher cell densities than the control cells (with pESC-Ura); and (ii) 5 % C11 alkane, BYL13 expressing the wild-type Pdr transcription factors (particularly Pdr3_wt_) had significantly higher cell densities, whilst BYL13 expressing the site-mutated Pdr transcription factors had modestly higher cell densities than the controls. The enhanced cell densities correspond with increased cell viability (Fig. 2b). However, there was no improvement in tolerance in BYL13 expressing the wild-type Pdr transcription factors against C10 alkane, or in BYL13 expressing any Pdr transcription factors against C8 or C9 alkanes. Expression of the representative transcription factors (Pdr1_mt1_ + Pdr3_mt_, and Pdr3_wt_) was confirmed by Western blots (Additional file 1: Figure S4). Furthermore, we performed quantitative PCR (qPCR) to understand the roles of *PDR* expression levels and site mutations in the tolerance improvement. Additional file 2: Tables S1 and S2 show that the C10 tolerance was attributed to the site mutations (Pdr1 F815S, and Pdr3 Y276H) regardless of the *PDR1*_*mt1*_*and PDR3*_*mt*_ expression levels. The results of the growth assays and expression analyses indicate that the expression of Pdr transcription factors improved yeast tolerance to C10 and C11 alkanes.

### Gene patterns in BYL13 expressing Pdr1_mt1_ + Pdr3_mt_ and Pdr3_wt_ in the presence of C10 and C11 alkanes

The improved alkane tolerance of yeast, conferred by the expression of Pdr1_mt1_ + Pdr3_mt_ or Pdr3_wt_, might be contributed to by perturbations made to the expression levels of the genes regulated by the Pdr transcription factors, such as ABC efflux pump genes, stress responsive genes, and genes involved in membrane modifications [22]. To examine this possibility, we studied the expression patterns of those target genes in the presence of C10 and C11 alkanes by qPCR.

First, to choose a suitable reference gene, we evaluated expression stability of five reference genes (*ACT1*, *ALG9*, *TAF10*, *UBC6*, and *TFC1*) by comparing their M values under the above conditions. Here, a lower M value stands for higher stability of gene expression [26, 27]. Additional file 2: Table S1 shows that *UBC6* gene had the lowest M value out of the five reference gene candidates under the conditions of Pdr expression and alkane exposure. Hence, *UBC6* was chosen as the reference gene for qPCR analyses.

**Fig. 3:**
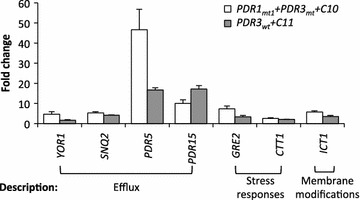
Relative gene expression levels under the regulation of Pdr transcription factors in the presence of C10 and C11 alkanes. In the induction medium containing 0.5 g/l galactose, BYL13 + Pdr1_mt1_ + Pdr3_mt_ cells were exposed to 1 % C10 alkane (PDR1_mt1_ + PDR3_mt_ + C10), and BYL13 + Pdr3_wt_ cells were exposed to 5 % C11 alkane (PDR3_wt_ + C11). The expression levels of genes at 24 h were normalized to those in controls (BYL13 + pESC-Ura + C10, BYL13 + pESC-Ura + C11) and the reference gene *UBC6*.* Error bars* SD from three biological replicates

Second, we compared expression levels of the target genes in BYL13 expressing Pdr1_mt1_ + Pdr3_mt_ (in the presence of C10 alkane) or Pdr3_wt_ (in the presence of C11 alkane) to those in BYL13 under exposure to C10 or C11 alkane. Figure 3 shows that, in BYL13 expressing either Pdr1_mt1_ + Pdr3_mt_ (C10) or Pdr3_wt_ (C11), (i) ABC efflux pump genes (i.e., *YOR1*, *SNQ2*, *PDR5*, and *PDR15*) were up-regulated by 4.2 to 46.6-fold (C10) and 1.6 to 17.1-fold (C11); (ii) cytosolic catalase gene *CTT1* was up-regulated by 2.1-fold (C10) and 2.6-fold (C11); and (iii) lysophosphatidic acid acyltransferase gene *ICT1* was up-regulated by 5.8-fold (C10) and 3.4-fold (C11). Considering the roles of the efflux pump genes in multi-drug resistance (MDR) [28], *CTT1* in ROS detoxification [29], and *ICT1* in membrane modifications [18], we hypothesized that intracellular alkane amount, ROS levels, the efficiency of alkane transport, and membrane damage might be involved in the increased alkane tolerance of BYL13 expressing Pdr1_mt1_ + Pdr3_mt_ or Pdr3_wt_. This hypothesis was investigated, as explained in the following sections.

### Alkane levels in BYL13 expressing Pdr1_mt1_ + Pdr3_mt_ and Pdr3_wt_

**Fig. 4:**
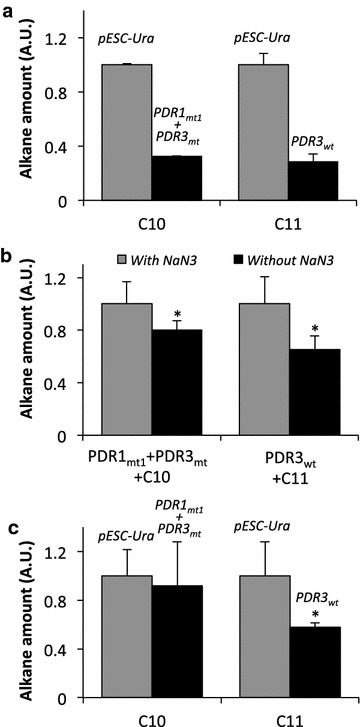
Analyses of intracellular alkane levels in BYL13 expressing Pdr transcription factors. **a** Upon exposure to C10 and C11 alkanes, respectively, intracellular alkanes were extracted, measured by GC, and quantified by normalizing areas of GC peaks to those of an internal standard (IS) n-dodecane as well as corresponding protein amount. **b** To identify involvement of ABC efflux pumps in reducing intracellular alkanes, intracellular alkanes were compared between BYL13 expressing Pdr transcription factors with and without NaN_3_. **c** To verify lower import of alkanes, intracellular alkanes were compared between BYL13 with pESC-Ura and expressing Pdr transcription factors in the presence of NaN_3_. Alkane amount in cells with pESC-Ura, and with NaN_3_ were set as 1. *Asterisks* significant difference (one-tailed Student *t* test, p < 0.05); *error bars* SD from three biological replicates

To investigate the effect of the expression of Pdr1_mt1_ + Pdr3_mt_ or Pdr3_wt_ on intracellular alkane levels, we quantified intracellular C10 and C11 alkanes by gas chromatography (GC) analyses. Figure 4a shows that (i) upon exposure to C10 alkane, the intracellular C10 alkane amount was reduced by 67 % in BYL13 expressing Pdr1_mt1_ + Pdr3_mt_; and (ii) upon exposure to C11 alkane, the intracellular C11 alkane amount was reduced by 72 % in BYL13 expressing Pdr3_wt_.

Given that C10 and C11 alkanes are not metabolized by *S. cerevisiae*, we hypothesized that the lower intracellular levels of alkanes in BYL13 expressing Pdr transcription factors could be due to alkanes efflux, reduced import, or both. First, to investigate the involvement of alkane efflux in reducing alkane accumulation, we exposed BYL13 expressing Pdr1_mt1_ + Pdr3_mt_ to C10 alkane and BYL13 expressing Pdr3_wt_ to C11 alkane. Following this, the ABC efflux pumps were deactivated, and the intracellular alkane amount with and without active ABC efflux pumps was compared. Here, NaN_3_ functions as a metabolic inhibitor that interferes with ABC transporters. The ABC transporters in the cells without NaN_3_ treatment are still active in alkane efflux. Under the condition of no NaN_3_ treatment, reduction of the intracellular alkane amounts is attributed to alkane efflux associated with the ABC efflux pumps, vise versa. Figure 4b shows that in comparison to the alkane levels in BYL13, C10 and C11 alkane levels in BYL13 with active ABC efflux pumps were reduced by 19.9 and 34.5 %, respectively. This result suggests significant reduction in intracellular C10 and C11 alkanes was contributed by ABC efflux pumps-associated alkanes export. Second, to verify whether lower import of alkanes contributed to reducing intracellular alkane levels, we added NaN_3_ to deactivate ABC efflux pumps, and then exposed the cells to C10 and C11 alkanes, and quantified intracellular alkanes. Upon the deactivation of the ABC transporters by NaN_3_, there is no ABC efflux pumps-associated alkane efflux during alkane import. Under this condition, lower intracellular alkane amounts are attributed to lower alkane import, vise versa. Figure 4c shows that, in comparison to the control without Pdr transcription factors, (i) C10 alkane amount was comparable in BYL13 expressing Pdr1_mt1_ + Pdr3_mt_, suggesting no difference in C10 alkane import despite the up-regulation of *ICT1* in BYL13 expressing Pdr1_mt1_ + Pdr3_mt_; and (ii) C11 alkane amount in BYL13 expressing Pdr3_wt_ was reduced by 42.2 %, suggesting lower import of C11, which is consistent with the up-regulation of *ICT1*. Hence, these results suggest that alkane efflux might contribute to C10 reduction, and both alkane efflux and low import to C11 reduction.

### ROS levels in BYL13 expressing Pdr1_mt1_ + Pdr3_mt_ and Pdr3_wt_ with C10 and C11 alkanes

**Fig. 5:**
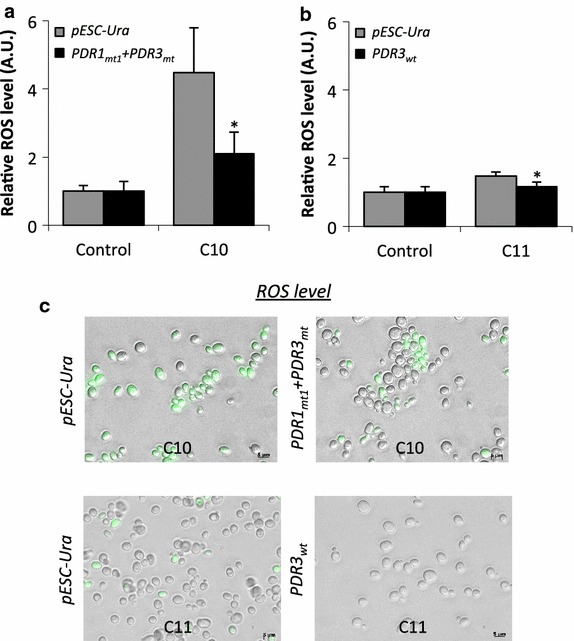
Quantification of ROS levels in BYL13 expressing Pdr transcription factors. **a** and **b** ROS levels upon exposure to C10 and C11 alkanes. The relative ROS levels of BYL13 without alkane were set to 1. **c** Comparison of fluorescence images, where stronger green fluorescence indicates higher ROS levels. *AU* arbitrary unit. *Asterisk* significant difference (one-tailed Student *t* test, p < 0.05);* error bars* SD from three biological replicates

ROS levels were quantified to investigate the effect of Pdr transcription factor expression on ROS production in the presence of alkanes. Figure 5a, b shows that C10 alkane enhanced ROS levels by more than fourfold whereas C11 alkane increased ROS levels by 1.5-fold. Further, in comparison to BYL13 carrying pESC-Ura, intracellular ROS was reduced by 53 % in BYL13 expressing Pdr1_mt1_ + Pdr3_mt_ in the presence of C10 alkane, and reduced by 21 % in BYL13 expressing Pdr3_wt_ in the presence of C11 alkane. The reduction of ROS in BYL13 expressing the Pdr transcription factors was further supported by our microscopy results. Figure 5c shows that, upon exposure to C10 alkane, over 90 % of the cells with pESC-Ura fluoresced in green, and only about 30 % of the cells with Pdr1_mt1_ + Pdr3_mt_ fluoresced in green. On the other hand, upon exposure to C11 alkane, 15 % of the cells with pESC-Ura fluoresced in green, and no cells with Pdr3_wt_ fluoresced in green. Here, more green cells and higher fluorescence intensities represent more ROS. The results of ROS quantification and microscopy suggest significant reduction of ROS in BYL13 expressing the Pdr transcription factors in the presence of C10 and C11 alkanes.

### Membrane integrity of BYL13 expressing Pdr1_mt1_ + Pdr3_mt_ and Pdr3_wt_ in the presence of C10 and C11 alkanes

The qPCR results in Fig. 3 show that *ICT1*, associated with membrane modifications, was up-regulated in BYL13 expressing Pdr transcription factors upon exposure to C10 and C11 alkanes, suggesting that membrane integrity might be affected upon exposure to alkanes [30]. Here, we aimed to study the membrane integrity of BYL13 expressing Pdr transcription factors in the presence of alkanes.

**Fig. 6:**
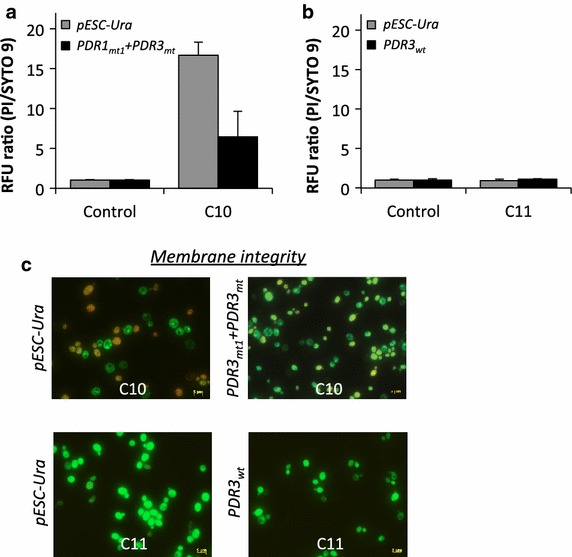
Assays of membrane integrity in BYL13 expressing Pdr transcription factors. **a** and **b** RFU ratios of PI and SYTO 9 upon exposure to C10 and C11 alkanes. The relative RFU ratios of BYL13 without alkane were set to 1. **c** Representative fluorescent images.* Error bars* SD from three biological replicates

To this end, we exposed the cells to C10 and C11 alkanes and stained the exposed cells with fluorescence nucleic acid stains PI and SYTO 9. Subsequently, we measured fluorescence signals and observed the cells under microscope. Figure 6a shows that relative fluorescence unit (RFU) ratios of PI to SYTO 9 were enhanced by 16.7-fold in BYL13 with pESC-Ura, and enhanced by 6.4-fold in BYL13 expressing Pdr1_mt1_ + Pdr3_mt_, upon exposure to C10 alkane as compared with those without alkane exposure. Moreover, in the presence of C10 alkane, the RFU ratio in BYL13 expressing Pdr1_mt1_ + Pdr3_mt_ was about 62 % lower than that with pESC-Ura, likely due to Ict1p-mediated membrane modifications in the presence of C10 alkane. However, Fig. 6b shows that, in the presence of C11 alkane, both BYL13 with Pdr3_wt_ and the control cells had comparable RFU ratios, suggesting intact cell membrane in the presence of C11 alkane.

The low RFU ratio suggests that BYL13 expressing Pdr1_mt_ + Pdr3_mt_ had less membrane damage than the control cells in the presence of C10 alkane, and the comparable ratios suggest no membrane damage to both BYL13 expressing Pdr3_wt_ and the control cells in the presence of C11 alkane, in line with the fluorescence microscopy images in Fig. 6c.

In this study, we improved yeast’s alkane tolerance by expressing wild-type or site-mutated Pdr transcription factors in *S. cerevisiaepdr1Δ pdr3Δ*, and provided the evidence that, in the most tolerant strains expressing Pdr transcription factors, (i) a series of genes (e.g., ABC efflux pump genes, *CTT1,* and *ICT1*) were up-regulated by C10 and C11 alkanes; (ii) intracellular alkane levels were reduced over 67 % due to alkane efflux and/or low import; and (iii) ROS levels were reduced over 21 %; and (iv) cell membrane damage was also reduced. However, expression of any Pdr transcription factors did not improve tolerance to C8 or C9 alkanes at toxic levels. The susceptibility of yeast to alkanes is associated with multiple factors such as alkane carbon-chain length, alkane concentration, and strain background. Additional file 1: Figure S2 shows that, more C8 and C9 alkanes were required to inhibit BYL13 with pESC-Ura than C10 alkane, although C8 and C9 alkanes are more toxic than C10 and C11 alkanes. This could be because C8 and C9 alkanes are more volatile than longer-chain alkanes. Furthermore, although Pdr1p and Pdr3p improved tolerance to C10 and C11 alkanes in BYL13, we found that Pdr1 *R821S* (Pdr1_mt2_) could not improve tolerance to C9 alkane, which is inconsistent with a previous study [21]. This discrepancy is likely due to difference of strain background between BYL13 and KK-211 used in the previous study. To demonstrate that strain background can affect cell tolerance towards alkanes, we expressed either Pdr1_mt1_ + Pdr3_mt_ or Pdr3_wt_ in BY4741, a parental strain of BYL13, and evaluated the cell growth in the presence of alkanes. Figure 2 and Additional file 1: Figure S5 show that BY4741 expressing Pdr transcription factors grew better than BYL13 expressing the same Pdr transcription factors upon exposure to C10 and C11 alkanes.

According to Mamnun and coworkers [22], Pdr1p and Pdr3p form homo- and hetero-dimers to mediate pleiotropic drug resistance in *S. cerevisiae*, and these homo- and hetero-dimers could show diverse transcriptional activity to their target genes involved in tolerance to alkanes. In line with the diversity of Pdr dimers and their transcriptional activity, Fig. 2 and Additional file 1: Figure S3 indicate discrepant tolerance conferred by the individual and co-expressed Pdr transcription factors.

Future efforts could be made to identify DNA-binding efficacy of Pdr1p- and Pdr3p-dimers as well as influences of various Pdr dimers on the transcriptome in response to alkanes, and to discriminate significance of each mechanism (alkanes efflux, membrane modifications, ROS reduction, and alleviation of membrane damage) in the Pdr transcription factors involving tolerance improvement towards alkane biofuels. In addition, a tool of global transcription machinery engineering (gTME) [19] can be applied to construct Pdr transcription factor libraries and obtain phenotypes of resistance against a wide spectrum of biochemical molecules.

## Conclusions

**Fig. 7:**
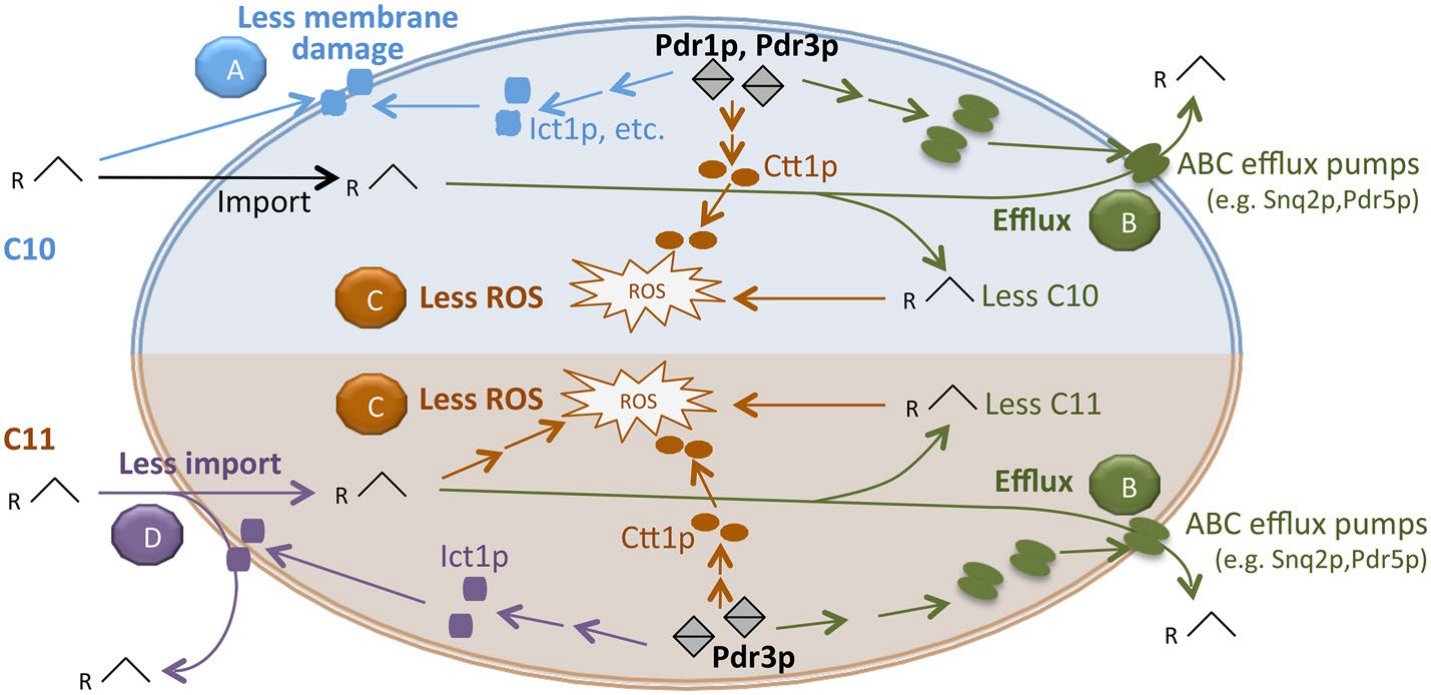
A schematic of proposed mechanisms on tolerance improvement towards C10 (*shaded* in *light blue*) and C11 (*shaded* in *light orange*) alkanes conferred by Pdr1p and Pdr3p. Pdr1p and Pdr3p improve tolerance to C10 alkane likely through reducing membrane damage (*A*, *blue*), alkane efflux (*B*, *green*), and reducing ROS production (*C*, *orange*); the tolerance to C11 alkane is improved likely through reducing C11 import due to potential membrane modifications (*D*, *purple*), as well as (*B*) and (*C*)

The site mutants Pdr1 F815S + Pdr3 Y276H (Pdr1_mt1_ + Pdr3_mt_) and the wild-type Pdr3p (Pdr3_wt_) improved the tolerance of BYL13 and BY4741 to C10 and C11 alkanes, respectively. We found that the mechanisms underlying Pdr1p- and Pdr3p-mediated tolerance are multilayered. As depicted in Fig. 7, we hypothesize that Pdr1p and Pdr3p regulate genes involved in alkane efflux (e.g., *SNQ2*, and *PDR5*), stress responses (e.g., *CTT1*) and membrane modifications (e.g., *ICT1*) in the presence of C10 and C11 alkanes. The tolerance to alkanes was improved through (i) reduced intracellular alkanes contributed by alkane efflux (C10 and C11) and lower alkane import (C11 alkane), (ii) decreased ROS production probably contributed by lower alkane accumulation (C10 and C11 alkanes) and Ctt1p-mediated ROS decomposition (C10 and C11 alkanes), and (iii) alleviated membrane damage contributed to by membrane modifications (C10 alkane). These findings provide valuable insights into engineering alkane-tolerant yeast for improved alkane productivity.

## Methods

### Strains, plasmids and growth conditions

Strains and plasmids used in this study are listed in Table 1. Yeast cells were grown at 30 °C in YPD (10 g/l Yeast Extract, 20 g/l Peptone, and 20 g/l Dextrose), minimal medium (6.7 g/l Yeast Nitrogen Base, 20 g/l Dextrose, and 1.92 g/l Yeast synthetic dropout medium supplements without uracil), or induction medium (6.7 g/l Yeast Nitrogen Base, 10 g/l D-raffinose, 1.92 g/l Yeast synthetic dropout medium supplements without uracil, and appropriate amount of D-(+)-galactose). *E. coli* was grown at 37 °C in Luria–Bertani (LB) broth. Antibiotics (200 μg/ml G418, or 100 μg/ml Ampicillin) and appropriate amount of n-alkanes were added if necessary.

**Table 1:** Strains, plasmids and *PDR* alleles used in this study

Strains, plasmids, and genes	Description	Sources
*S. cerevisiae* strains		
BY4741	*MATa; his3∆1; leu2∆0; met15∆0; ura3∆0*	ATCC201388
BYL1K	*pdr1::kanMX* derived from BY4741	This study
BYL13K	*pdr3::kanMX* derived from BYL1K	This study
BYL13	*pdr1∆ pdr3∆*, KanMX-free BYL13K	This study
Plasmids		
pESC-Ura	Expression vector, Ura^+^	Agilent
pRS42K	Expression vector, Kan^+^	Euroscarf
pSH47	A plasmid for marker rescue, Ura^+^	Euroscarf
pUG6	A plasmid with a KanMX gene cassette, Amp^+^	Euroscarf
*PDR1 and PDR3 alleles*		
*PDR1*_*wt*_	Wild-type *PDR1*	This study
*PDR1*_*mt1*_	A site mutant of *PDR1* with amino acid substitution of F815S	This study, [24]
*PDR1*_*mt2*_	A site mutant of *PDR1* with amino acid substitution of R821S	This study, [21]
*PDR1*_*mt1*+*2*_	A site mutant of *PDR1* with amino acid substitution of both F815S and R821S	This study
*PDR3*_*wt*_	Wild-type *PDR3*	This study
*PDR3*_*mt*_	A site mutant of *PDR3* with amino acid substitution of Y276H	This study, [23]

### Disruption of *PDR1* and *PDR3*

Gene disruption was carried out as previously described [31] using primers listed in Additional file 2: Table S3. First, gene disruption cassettes of *loxP*-*Kan*-*loxP* were amplified from pUG6 and transformed into *S. cerevisiae* BY4741. Colonies were screened on YPD agar plates containing 200 μg/ml G418. Subsequently, pSH47 was transformed into a single mutant BYL1K, and transformants were screened on minimal medium (Ura^−^) plates, followed by Cre/loxP-mediated marker removal in galactose-induction medium. Selection for loss of the marker was performed in YPD containing 1 mg/ml 5-fluoro-orotic acid (Thermo Scientific). Finally, marker-free *pdr1Δ* (BYL1) was used as a parental strain for disruption of *PDR3*.

### Gene cloning and expression

With *S. cerevisiae* BY4741 genomic DNA as a template, wild-type *PDR1* and *PDR3* were amplified using iProof High-Fidelity DNA Polymerase (Biorad) and gene-specific primers (Additional file 2: Table S3). A-tailing of PCR products was performed with Taq polymerase (New England Biolabs). The PCR products were purified, cloned into pGEM-T (Promega) and confirmed by sequencing. Subsequently, correct recombinant plasmids were used as templates for site mutagenesis using Quick Change Site-Directed Mutagenesis Kit (Stratagene) according to a provided manual. Further, site mutations were confirmed by sequencing. Thereafter, wild-type and site mutants of *PDR1* and *PDR3* were amplified and co-transformed with linearized pESC-Ura fragments into BYL13 by an in vivo DNA assembly method [32]. Finally, transformants were screened on minimal medium agar plates (Ura^−^) and characterized by PCR and sequencing. Here, *PDR1* and its site mutants were cloned into multiple cloning site 2 (MCS2) under control of a galactose-inducible promoter *P*_*GAL1*_, while *PDR3* and its site mutant were cloned into MCS1 under control of another galactose-inducible promoter *P*_*GAL10*_. These *PDR* genes in MCS1 and MCS2 were individually expressed or co-expressed under induction by galactose (Fig. 1).

### Western blotting

Pdr proteins were induced in the induction medium containing 0.5 g/l galactose for 24 h. Fifty milliliters of cells were collected and resuspended in ice-cooled lysis buffer (50 mM Tris–HCl pH7.9, 0.6 M sorbitol) added with protease inhibitor and 0.3 g acid-washed glass beads (diameter 425–600 μm). Cells were disrupted using FastPrep-24 at 6 m/s for 30 s × 10 cycles (MP Biomedicals, USA). Samples were chilled on ice for 5 min during each interval. Cell lysate was centrifuged at 4 °C to separate soluble and insoluble proteins. The insoluble part was dissolved with 8 M urea. The obtained protein samples were separated by SDS-PAGE, transferred onto nitrocellulose membrane, and hybridized with anti-Myc (for Pdr1p) and anti-Flag (for Pdr3p) antibodies (Abcam). Protein bands with positive signal were detected using SuperSignal West Pico Chemiluminescent Substrate (Thermo Scientific).

### Tolerance tests

First, we determined conditions of protein induction. In induction media with 20 g/l galactose, we measured growth curves of BYL13 expressing Pdr transcription factors, and a strain with minimal growth was selected to optimize galactose concentrations. Subsequently, the selected strain was grown in induction media with galactose (0.5 g/l, 5 g/l, and 20 g/l). Every 12 h, 100 μl cell culture was sampled to measure cell densities (OD_600_) by a BioTek microplate reader, and galactose concentration which gave low growth inhibition was chosen for tolerance tests. Second, we determined conditions of alkanes exposure as follows. BYL13 with pESC-Ura was grown in induction media with galactose at the previously determined concentration, and exposed to alkanes (v/v, C8: 2, 5, 10 %, C9: 1, 2, 5 %, C10: 0.5, 1, 5, and C11: 5, 10, 20 %). Based on growth curves, minimum concentrations of alkanes that inhibited BYL13 with pESC-Ura were selected for tolerance tests. Third, to test tolerance of cells with Pdr transcription factors against alkanes, we grew the cells in induction media under the determined conditions for protein induction and alkanes exposure. Strains showing the highest cell densities were selected for further analyses.

### Total RNAs extraction and quantitative PCR analyses

BYL13 expressing Pdr1 F815S/Pdr3 Y276H (Pdr1_mt1_ + Pdr3_mt_) and wild-type Pdr3p (Pdr3_wt_) were exposed to 1 % C10 and 5 % C11, respectively, for 6 h. Thereafter, the treated cells were collected, and cell wall was disrupted by Lyticase (Sigma). Total RNAs were extracted using an RNeasy Mini Kit (Qiagen) according to a provided manual. Single-stranded cDNAs were synthesized from 1 µg total RNA using a RevertAid First Strand cDNA Synthesis Kit (Thermal Scientific). With an equal amount of cDNAs, qPCR was performed using gene-specific primers (Additional file 2: Table S3) and Biorad SsoFast EvaGreen Supermix, and fluorescence signals were detected and analyzed by a Biorad iQ5 optical system. Expression stability of five reference gene candidates (*ACT1*, *ALG9*, *TAF10*, *TFC1*, and *UBC6*) was evaluated subjective to M value [26, 27]. Finally, gene expression data were normalized to a reference gene *UBC6* and controls. Here, samples from BYL13 with pESC-Ura and alkanes exposure were used as controls.

### Alkane extraction and quantification

Alkanes were extracted by a chloroform–methanol extraction method [33] with modifications. First, BYL13 expressing Pdr1_mt1_ + Pdr3_mt_ and BYL13 expressing Pdr3_wt_ were exposed to 1 % C10 and 5 % C11, respectively. Thereafter, the alkane-treated cells were collected, washed with 50 mM Tris–Cl (pH 7.5), and resuspended in 0.5 ml chloroform–methanol mixture (v/v: 2/1). With 0.01 % n-dodecane (C12) and 0.3 g glass beads per sample, cells were lysed by FastPrep-24, and lysate was separated by centrifugation at a top speed for 10 min at 4 °C. Supernatant was collected, and alkanes were extracted by adding appropriate amount of chloroform and 50 mM Tris–Cl (pH7.5). Finally, chloroform phases containing alkanes were injected into a GC 7890A system and analyzed under conditions as follows: with a HP-5 column (Agilent Technologies), oven temperature started at 80 °C, held for 1 min, ramped at 20 °C/min until 180 °C, and held for 2 min; FID detector temperature remained at 275 °C. A mixture of n-alkanes (10 ppm each) was used as a standard. Finally, areas of GC peaks were normalized to an internal standard and corresponding protein amount.

To investigate involvement of alkane efflux in reducing intracellular alkane amount, BYL13 expressing Pdr1_mt1_ + Pdr3_mt_ and BYL13 expressing Pdr3_wt_ were grown until log phases and exposed to 1 % C10 and 5 % C11, respectively, for 1 h. Cells were washed and divided into two equal aliquots. The first aliquot was treated with 1 mM NaN_3_ for 80 min, to deactivate activities of ABC transporters [34]. Here, NaN_3_ functions as a metabolic inhibitor that interferes with ABC transporters by decreasing the amount of ATP generated by mitochondria. The second aliquot was incubated without NaN_3_ for 80 min. Finally, alkanes were extracted and quantified.

To investigate involvement of alkanes import in reducing intracellular alkane amount, BYL13 expressing Pdr transcription factors (i.e., Pdr1_mt1_ + Pdr3_mt_, and Pdr3_wt_) and BYL13 with pESC-Ura (control), respectively, were grown in an alkane-free induction medium until log phases and treated with 1 mM NaN_3_ for 20 min to deactivate activities of ABC transporters [34]. An equal amount of the NaN_3_-treated cells were subsequently exposed to 1 % C10 and 5 % C11, respectively, for 1 h. After exposure, alkanes were extracted and quantified.

### ROS quantification and membrane integrity analyses

BYL13 expressing Pdr1_mt1_ + Pdr3_mt_ and BYL13 expressing Pdr3_wt_ at log phases were exposed to 1 % C10 and 5 % C11, respectively, for 6 h. Thereafter, the alkane-treated cells were collected and stained by CellROX^®^ Green Reagent (Life Technologies) for ROS analyses, and by SYTO 9 and propidium iodide (PI) (Life Technologies) for membrane integrity analyses. Further, fluorescence signals from CellROX^®^ Green Reagent were measured by a TECAN Infinite 200 microplate reader at wavelength of 485 nm (Excitation, Ex)/535 nm (Emission, Em), while fluorescence signals from PI and SYTO 9 were measured at wavelength of 535 nm (Ex)/590 nm (Em) and 485 nm (Ex)/535 nm (Em), respectively. The acquired fluorescence signals were normalized to cell densities (OD_600_) if necessary. In addition, the stained cells were observed at wavelength of 535 nm (Ex)/590 nm (Em) and 470 nm (Ex)/525 nm (Em) under a Zeiss Axio Scope.A1 microscope.

## Additional files

10.1186/s13068-015-0411-z Additional figures. **Figure S1.** Growth inhibition by expression of the site-mutants of Pdr transcription factors, and determination of suitable galactose concentrations.** Figure S2.** Determination of alkane exposure concentrations. **Figure S3.** Growth of BYL13 expressing Pdr transcription factors in the presence of alkanes.** Figure S4. **Western blotting of Pdr proteins in BYL13. **Figure S5.** Tolerance of BY4741 expressing Pdr transcription factors against C10 and C11 alkanes. 
10.1186/s13068-015-0411-z Additional tables.**Table S1.** M values of reference gene candidates derived from qPCR data. **Table S2.** Fold changes of PDR genes expressed in BYL13 stains as compared to BY4741. **Table S3.** Primers used in this study.

## Abbreviations

PDR: pleiotropic drug resistance
Wt: wild type
Mt: mutant
C8: n-octane
C9: n-undecane
C10: n-decane
C11: n-undecane
C12: n-dodecane
ROS: reactive oxygen species
ABC: ATP-binding cassette
MCS: multiple cloning sites
PI: propidium iodide
qPCR: quantitative PCR
RFU: relative fluorescence unit
