# Supplementary material for: Engineering transcription factors to improve tolerance against alkane biofuels in Saccharomyces cerevisiae
Source: Biotechnol Biofuels. 2015 Dec 30;8:231. doi: 10.1186/s13068-015-0411-z (PMC4696261; doi:10.1186/s13068-015-0411-z)
Supplement: Supplementary file 1 — 10.1186/s13068-015-0411-z Additional figures. Figure S1. Growth inhibition by expression of the site-mutants of Pdr transcription factors, and determination of suitable galactose concentrations. Figure S2. Determination of alkane exposure concentrations. Figure S3. Growth of BYL13 expressing Pdr transcription factors in the presence of alkanes. Figure S4. Western blotting of Pdr proteins in BYL13. Figure S5. Tolerance of BY4741 expressing Pdr transcription factors against C10 and C11 alkanes. [file 13068_2015_411_MOESM1_ESM.pdf]

Figure S1. Growth inhibition from expressing the site-mutants of Pdr transcription factors and determination of suitable galactose concentration. (A) Growth curves under induction by 20 g/l galactose. Control, BYL13 with pESC-Ura, Pdr1$_{mt1}$ (Pdr1 *F815S*), Pdr1$_{mt2}$ (Pdr1 *R821S*), Pdr1$_{mt1+2}$ (Pdr1 *F815S R821S*), Pdr3$_{mt}$ (Pdr3 *Y276H*). (B) Growth inhibition from the co-expressed Pdr1$_{mt1+2}$ and Pdr3$_{mt}$ under induction by galactose (Gal) at concentrations of 0.5 g/l, 5 g/l, and 20 g/l. Growth of BYL13 with pESC-Ura at each time point was set to 100%. Error bars, SD from three biological replicates.

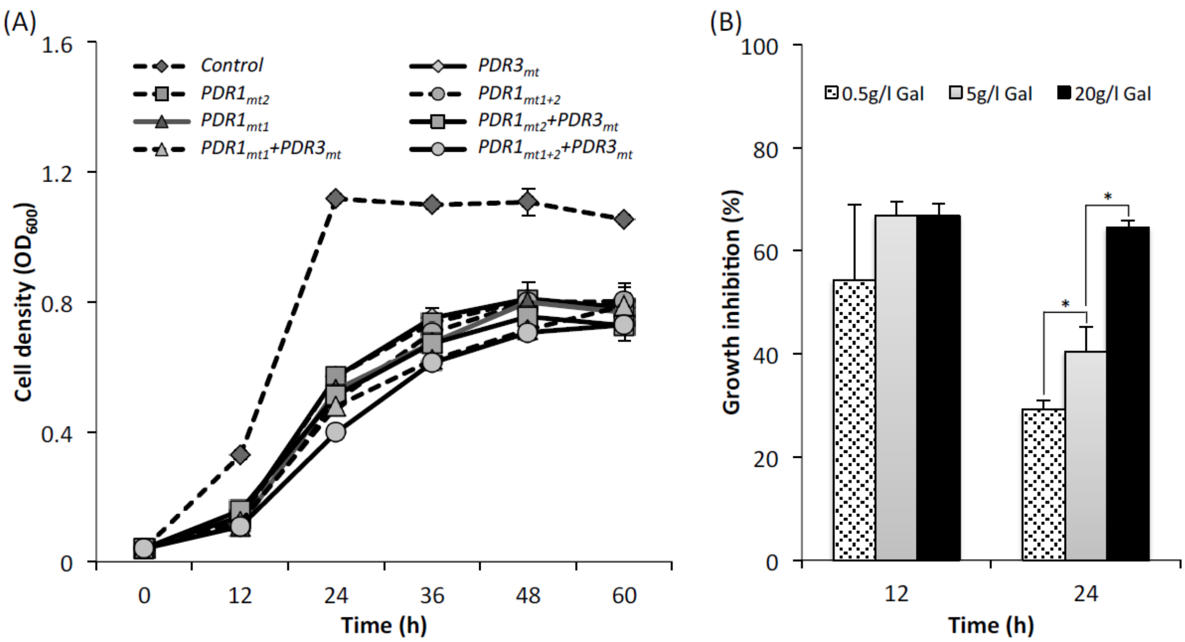


Figure S2. Determination of alkane exposure concentrations. BYL13 with pESC-Ura was incubated in induction media added with 0.5 g/l galactose, without alkane (A) and with different amounts of C8 (B), C9 (C), C10 (D), and C11 (E) alkanes. Error bars, SD from three biological replicates.

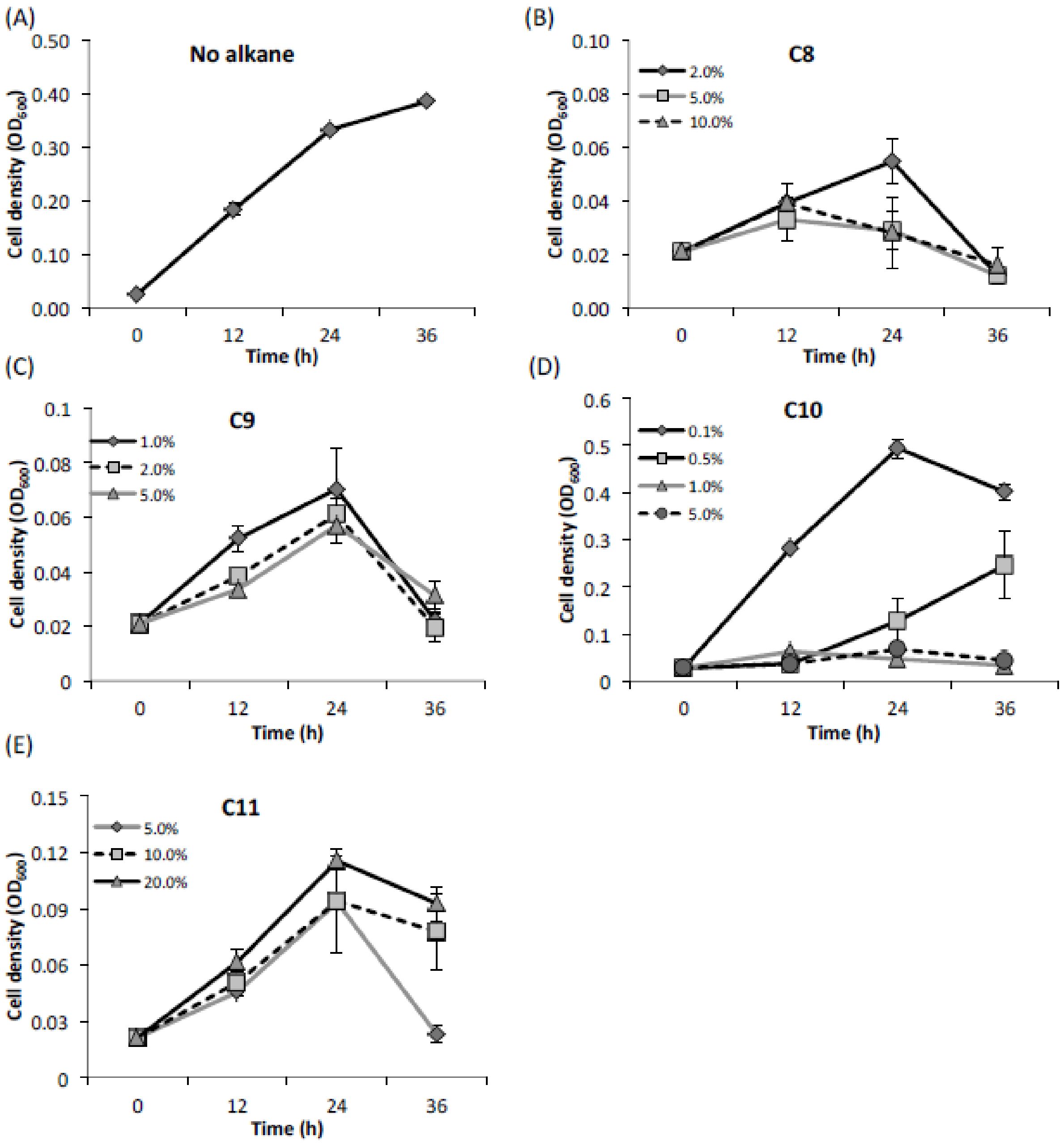


Figure S3. Growth of BYL13 expressing Pdr transcription factors in the presence of alkanes. Under induction by 0.5 g/l galactose, BYL13 expressing Pdr transcription factors were exposed to 1% C10 and 5% C11, respectively. Wild type (WT) and site-mutants (MT) of Pdr1p and Pdr3p are indicated. Control, BYL13 with pESC-Ura. Error bars, SD from three biological replicates.

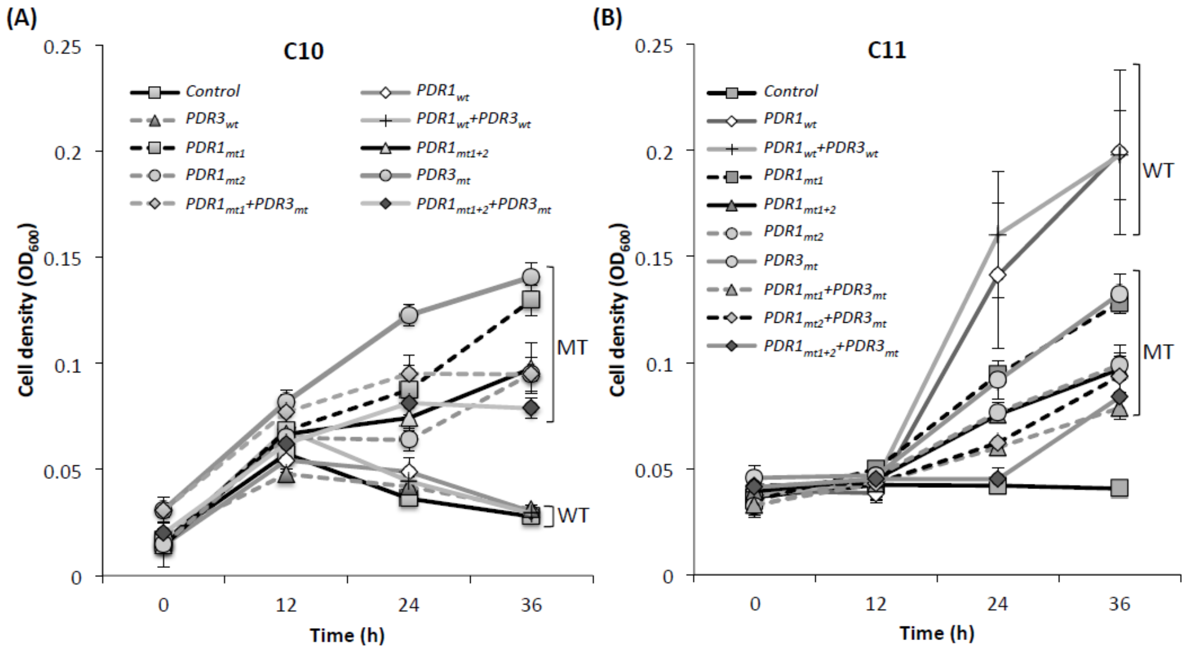


Figure S4. Western blotting of Pdr proteins in BYL13. $Pdr1_{mt1}$+$Pdr3_{mt}$ (A) and $Pdr3_{wt}$ (B) were induced by 0.5 g/l of galactose. Positive bands are indicated by arrows, with sizes of about 122 kDa (Pdr1) and 113 kDa (Pdr3). Asterisks, mutation sites. IS, insoluble form, S, soluble form.

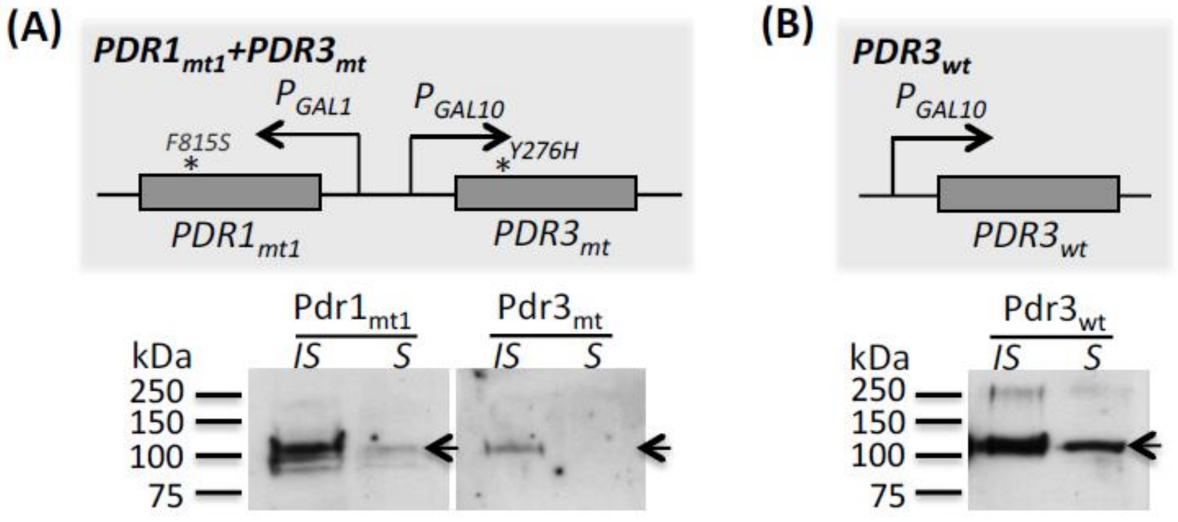


Figure S5. Tolerance of BY4741 with Pdr transcription factors against C10 and C11 alkanes. Under induction by 0.5 g/l galactose, BY4741 with pESC-Ura, $Pdr1_{mt1}$+$Pdr3_{mt}$, and $Pdr3_{wt}$ were exposed to 1% C10 (diamond) and 5% C11 (square), respectively. Cell densities ($OD_{600}$) were measured and compared every 12 h. Error bars, SD from three biological replicates.

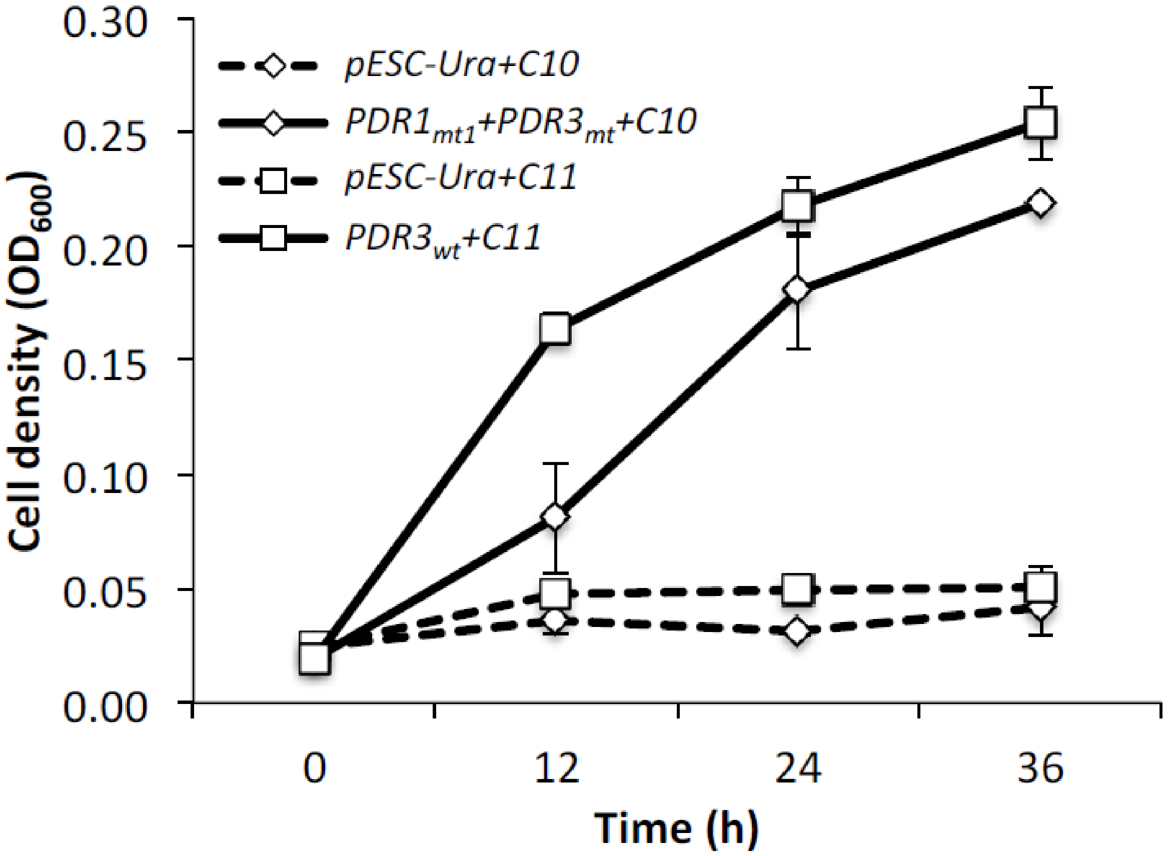

0.30
0.25
0.20
0.15
0.10
0.05
0.00
Cell density ($OD_{600}$)
pESC-Ura+C10
$PDR1_{mt1}$+$PDR3_{mt}$+C10
pESC-Ura+C11
$PDR3_{wt}$+C11
0
12
24
36
Time (h)
